# Factors associated to unrelieved pain in a Morrocan Emergency Department

**DOI:** 10.1186/1755-7682-7-48

**Published:** 2014-11-08

**Authors:** Maha louriz, Jihane Belayachi, Bouchra Armel, Tarek Dendane, Khalid Abidi, Naoufel Madani, Aicha Zekraoui, Abdellatif Belabes Benchekroun, Amine Ali Zeggwagh, Redouane Abouqal

**Affiliations:** Medical Emergency Department, Ibn Sina University Hospital, 10000 Rabat, Morocco; Medical Intensive Care Unit, Ibn Sina University Hospital, 10000 Rabat, Morocco; Surgical Emergency Department, Ibn Sina University Hospital, 10000 Rabat, Morocco; Laboratory of Biostatistics, Clinical and Epidemiological Research, Faculté de Médecine et Pharmacie- Université Mohamed V Souissi, 10000 Rabat, Morocco

**Keywords:** Emergency department, Pain, Pain relief, Unrelieved pain

## Abstract

**Background:**

In the light of the impact that pain has on patients, emergency department (ED) physicians need to be well versed in its management, particularly in its acute presentation. The goal of the present study was to evaluate the prevalence of unrelieved acute pain during ED stay in a Moroccan ED, and to identify risk factors of unrelieved pain.

**Methods:**

Prospective survey of patients admitted to the emergency department of Ibn Sina teaching university hospital in Rabat (Morocco). All patients with acute pain over a period of 10 days, 24 hours each day were included. From each patient, demographic and clinical data, pain characteristics, information concerning pain management, outcomes, and length of stay were collected. Pain intensity was evaluated both on arrival and before discharge using Numerical Rating Scale (NRS). Comparison between patient with relieved and unrelieved pain, and factors associated with unrelieved pain were analyzed using stepwise forward logistic regression.

**Results:**

Among 305 patients who complained of acute pain, we found high levels of intense to severe pain at ED arrival (91.1%). Pain intensity decreased at discharge (46.9%). Unrelieved pain was assessed in 24.3% of cases.

Patients with unrelieved pain were frequently accompanied (82.4% vs 67.1%, p *=* 0.012), and more admitted daily than night (8 am-20 pm: 78.4% vs 64.9%; 21 pm-7 am: 21.6% vs 35.1%, p = 0.031), and complained chiefly of pain less requently (56.8% vs 78.8%, p<0.001). They had progressive pain (73% vs 44.2%, p<0.001), and had a longer duration of pain before ED arrival (72-168 h: 36.5% vs 16.9%; >168 h: 25.5% vs 17.7%, p<0.001).

In multivariate analysis, predictor factors of unrelieved pain were: accompanied patients (OR = 2.72, 95% CI = 1.28- 5.76, p = 0.009), pain as chief complaint (OR = 2.32, 95% CI = 1,25-4.31, p = 0.007), cephalic site of pain (OR = 6.28, 95% CI = 2.26-17.46, p<0.001), duration of pain before admission more than 72 hours (72-168 h (OR = 7.85, 95% CI = 3.13-25.30, p = 0.001), and >168 h (OR = 4.55, 95% CI = 1.77-14.90, p = 0.02).

**Conclusion:**

This study reported high levels of intense to severe pain at ED arrival. However, one quarter patients felt on discharge from the ED that their pain had not been relieved. The relief of pain in ED depend both sociodemographic, clinical, and pain characteristics factors.

## Introduction

Studies of pain management began to appear in the emergency medicine literature around 1990. Most are retrospective studies of patients with acute conditions that are perceived by most to be painful. Although these studies differ in design and population survey, they jointly document a historical litany of under relief of pain across a broad demographic range of patients and practice settings [1–12]. This substantial increase in emergency department (ED) research focusing on pain management reflects the desire of health care professionals to optimize and improve the management of pain [13, 14]. In the light of the impact that pain has on patients, ED physicians need to be well versed in its management, particularly in its acute presentation. Thus, Practical and time-sensitive approaches to pain and pain management will continue to be a challenge to enact and enforce in our ED.

Unrelieved pain remains a global health problem. The costs of pain in USA was around US$100–200 billion a year. This is equivalent to what it has been costing every year of the Iraq war for USA ($100–150 billion a year) [15]. Thus, the difference between; what could be done to relieve pain and what is being done in developing countries; is big. Limited facilities for pain treatment, and poor access to drugs for pain relief are contributing factors. Thus, enthusiasm for pain education and clinical training in developing countries has grown [16].

Morocco is a country of 32 million inhabitants situated in North Africa. The health budget corresponds to 1.1 percent of gross domestic product and 5.5 percent of the central government budget [17]. Morocco has inadequate numbers of physicians (0.5 per 1,000 people) and hospital beds (1.0 per 1,000 people) besides poor access to water (82% of the population) and sanitation (75% of the population). The health care system includes 122 hospitals, 2.400 health centers, and 4 university clinics, but they are poorly maintained with inadequate capacity to meet the demand for medical care [17]. Only 24.000 beds are available for 6 million patients seeking care each year, including 3 million emergency cases. Morocco has two major health sectors, public and private; the latter is said to be complementary rather than competitive. Patients may choose whether to attend primary or secondary, public or private care however, there is also a semi public sector [17]. The majority of Moroccans in employment pay for health insurance, which covers most, but not all, of health expenses within the public and private sectors. This health insurance remains valid even after pension.

Pain is an “overwhelming situation” for the Moroccan health care professionals. Several factors explain this situation; lack of pain assessment, lack of training and resources, inappropriate opioid myths and practices, lack of involvement of nurses and families. Therefore, protocol for pain assessment and management that would take into consideration the available resources is a great need [18].

To our knowledge, there is no study evaluating acute pain experience in Moroccan ED. In this survey, we aimed to evaluate the prevalence of unrelieved acute pain during ED stay in a Moroccan ED and to identify factors associated with unrelieved pain.

## Methods

### Study design

This was a cross-sectional survey with prospective data collection.

### Study area

The study was performed at ED of Ibn Sina University hospital in Rabat. Ibn Sina university hospital in Rabat is the referral for habitants in Western-North of Morocco, it is a 1028 bed tertiary – stage hospital that opened in 1955. The bed occupancy rate is of 76% to 85%. The hospital comprises 24 departments (12 surgical, 9 medicals, and 3 intensive care). This hospital provides all major adult medical and surgical departments except gynecology-obstetric, ophthalmology, otolaryngology, and oncology. The mean of ED visits per day is 176. The ED comprises two units (medical and surgical). Short stay admission in ED does not exceed 72 hours. The study was approved by the local ethics committee and informed consent was obtained from all patients.

### Duration of study

Data were collected during 10 days, 24 hours per day, from 6 to 16 November 2008.

### Study population

We included all consecutive patients hospitalized in the ED during the study period, aged 16 years or over, and who were able to self-assess pain.

### Exclusion criteria

Patients were excluded from the study for any of the following:

- Chronic pain: Persistent pain for more than 3 months
- Altered mental state
- Neuropsychiatric diseases
- life-threatening: Vital distress, respiratory distress
- Substance abuse: Drug abuse with illicit drugs and licit drugs
- Inability to communicate ; language barrier
- Refusal to participate in the study

### Data collection

Standardized collection of demographic and clinical data was performed by a trained research assistant using data collection forms. The data collection forms were structured in two parts. The first part contains questions for information on the characteristics of patients with acute pain hospitalized in ED.

Acute pain was defined as pain of recent onset and probable limited duration (International Association for the Study of Pain [IASP] definition). For the purposes of this study, pain was defined as chronic if its duration was longer than three month.

Collected data from each patient included age and gender, type of emergency (medical or surgical disease), educational level (none, primary, secondary, higher), marital status (unmarried/married), arrival hour to ED (8 am-20 pm, 21 pm-7 am), and if patient was accompanied in the ED (yes, no). Accompanied patients were defined as all patients who had individuals (relatives or friends) with them during their stay in the ED. Acute pain characteristics included; site of pain (abdominopelvic, thoracic, cephalic, musculoskeletal, lumbar, multifocal), if pain is chief complaint (yes, no), mode of appearance (sudden or progressive), duration of pain before admission (per hours) (<6 h, 6-72 h, 72-168 h, >168 h), pain intensity on arrival and before discharge.

The second part contains questions about information on the characteristics of pain management including: recent use of analgesics before ED admission to relieve the current pain (yes, no), delay of care (per minutes) (<30 min, 30-60 min, 60-120 min, >120 min), nature of pain management (analgesics, specific treatment, immobilization, psychologic approach), patient outcomes (discharge to home or transfer to an hospital ward), and length of stay in ED (per hours). An analgesic was defined as any medicine prescribed to reduce pain such as acetaminophen, a nonsteroidal anti-inflammatory drug, an opioid, an antacid for abdominal pain, or nitrates for chest pain.

### Protocol of pain assessment

Patients self-assessed pain using a 10-point numerical rating scale (NRS) with 0 no pain, and 10 worst possible pain) both on arrival; and before discharge [19, 20]. Intensity of pain was defined as mild if the NRS score was ≤3/10, as intense if the NRS score was >3/10 and <6/10, and as severe if the NRS score was ≥6/10.

Considering the different causes and types of pain, as well as its nature and intensity, relief of pain requires an interdisciplinary approach. The elements of this approach include treating the underlying cause of pain, pharmacological and non pharmacological therapies, and some invasive procedures. We assessed pain progression by calculating the difference between NRS score on arrival and at discharge. If the difference was ≥2 or over, pain intensity had increased, if the difference was between -1 and 1, pain intensity was stable and if the difference ≤ -2, pain intensity had decreased. When the pain intensity decreased or when patients no longer complained of pain on discharge pain was considered as relieved. When the pain intensity had increased or was stable pain was considered as unrelieved [10].

### Statistical analysis

Qualitative variables were presented as number and percentages. Quantitative variables were presented as mean ± standard deviation for variables with normal distribution, and as median and interquartile range (IQR) for variables with skewed distributions. The normality of the distribution was tested by the Kolmogorov-Smirnov test with Lilliefors correction. Univariate analysis was used to compare sociodemographic, and pain characteristics between “unrelieved” and “relieved” patients. Statistical differences between groups were evaluated by the chi-square test, and Fisher exact test (<5 expected events in a cell of the contingency table) for categorical variables. Comparison of group differences for continuous variables was carried out by Student test or the Mann_Whitney U test. Variables with p value lower than 0.2 in the univariate analysis were tested in the multivariate analysis. Multivariate analysis was performed using stepwise forward logistic regression models. Associations are expressed as odds ratios (ORs) with 95% confidence intervals (95% CIs). Calibration is defined as the agreement between individual probabilities and actual outcomes. The Hosmer-Lemeshow goodness of fit statistic was used to evaluate calibration of each predictive model. Discrimination is defined as the power to distinguish between unrelieved” and “relieved” patients and was assessed by calculating the area under the receiver operating characteristic (aROC) curve. All tests were two-tailed and statistical significance was set at p <0.05. Statistical analyses were performed with SPSS 17.0 for Windows (SPSS, Inc., Chicago, IL, USA).

## Results

**Figure 1:**
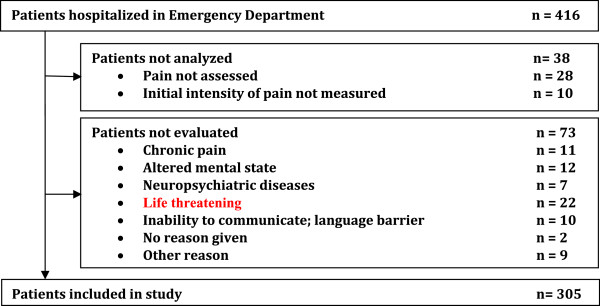
**Shart flow of patient hospitalized in the Emergency Department of a Moroccan university hospital.**

During the 10 days study period, among the 416 patients who were hospitalized in ED, 38 (9%) were not analyzed, and 73 (17.5%) were excluded. Analysis was therefore conducted on the remaining 305 patients (Figure 1).

Acute pain was the chief complaint in 73.4% of cases, appeared suddenly in 49% of cases. At the first interview, pain was categorized as intense to severe in 91.1% of cases. The rate of analgesics use was of 78.1%. At discharge, the intensity of pain decreased in 46.9% of cases. Characteristics of patient with acute pain are reported in Table 1. Patients with unrelieved pain compared to those with relieved pain were more frequently accompanied (61(82.4%) vs 155 (67.1%), p = 0.012), had pain as chief compliant less frequently (42 (56.8%) vs 182 (78.8%), p < 0.001), were admitted more frequently daily than night hours (8 am-20 pm: 58 (78.4%) vs 150 (64.9%); 21 pm-7 am: 19 (21.6%) vs 81 (35.1%), p = 0.031), their pain appeared progressively (54 (73%) vs 102 (44.5%), p < 0.001), and long before the ED visit (72-168 h: 27 (36.5%) vs 39 (16.9%); >168 h:19 (25.5%) vs 41 (17.7%), p < 0.001). Comparison of pain characteristics and pain management practices between patients with unrelieved and relieved pain in ED are reported in Table 2.

**Table 1:** **Characteristics of patient with acute pain** (**n** = **305**)

characteristics	n (%)
**Age** (years)	
≤30	85 (27.9)
30-40	65 (21.3)
40-60	104 (34)
>60	51 (16.7)
**Gender**	
Male	177 (58)
Female	128 (42)
**Accompanied patient**	
Yes	216 (70.8)
no	89 (29.2)
**Educational level**	
None	161 (52.8)
Primary	65 (21.3)
Secondary	65 (21.3)
Higher	14 (4.6)
**Marital status**	
Unmarried	133 (43.6)
Married	172 (56.4)
**Type of emergency**	
Medical	125 (41)
Surgical	180 (59)
**Pain as chief complaint**	
Yes	224 (73.4)
No	81 (26.6)
**Arrival hour to ED**	
8 pm -20 am	208 (68.2)
21 am -7 pm	97 (31.8)
**Site of pain**	
Abdominopelvic	98 (32.1)
Musculoskeletal	60 (19.7)
Thoracic	59 (19.3)
Cephalic	42 (13.8)
Lumbar	19 (6.2)
Multifocal	27 (8.9)
**Mode of appearance**	
Sudden	149 (48.9)
Progressive	156 (51.1)
**Duration of pain before admission** **(Hours),**	
<6	82 (26.9)
6-72	97 (31.8)
72-168	66 (21.6)
>168	60 (19.7)
**Medication before admission**	
Yes	83 (26.4)
no	231 (73.6)
**Delay of care** **(Hours),**	
<30	125 (41)
30-60	102 (33.4)
60-120	61 (20)
>120	17 (5.6)
**Pain intensity**	
Mild	28 (8.9)
Intense	58 (18.5)
Severe	228 (72.6)
**Nature of pain management**	
Analgesics	246 (78.1)
Specific treatment	193 (61.2)
Immobilisation	73 (23.1)
Psychologic approach	23 (7.3)
**Pain intensity on ED discharge**	
Mild	162 (53.1)
Intense	90 (29.5)
Severe	53 (17.4)
**Pain relief**	
Unrelieved pain	74 (24.5)
Relieved pain	231 (75.7)
**Length of stay** **(Hours),** **median** **(IQR)**	48 [48–72]

Length of stay was expressed as median and interquartile range (IQR); *ED*: emergency department.

**Table 2:** **comparison of pain characteristics and pain management practices between patients with unrelieved pain** (***n*** = **74**) **and relieved pain** (***n*** = **231**) **in the Emergency Department**

Characteristics	Relieved pain(n = 231)	Unrelieved pain (n = 74)	p value
Age (years),			0.16
≤30	69 (29.9)	16 (21.6)	
30-40	53 (22.9)	12 (16.2)	
40-60	73 (31.6)	31 (41.9)	
>60	36 (15.6)	15 (20.3)	
Gender			0.41
Male	131 (56.7)	46 (62.2)	
Female	100 (43.3)	28 (37.8)	
Accompanied patient			0.012
Yes	155 (67.1)	61 (82.4)	
No	76(32.9)	13 (17.6)	
Educational level			0.062
None	113 (48.9)	48 (64.9)	
Primary	52 (22.5)	13 (17.6)	
Secondary	56 (24.2)	9 (12.2)	
Higher	10 (4.3)	4 (5.4)	
Marital status			0.15
Unmarried	106 (45.9)	27 (36.5)	
Married	125 (54.1)	47 (63.5)	
Type of emergency			0.47
Medical	92 (39.8)	33 (44.6)	
Surgical	139 (60.2)	41 (55.4)	
Pain as chief complaint			< 0.001
Yes	182 (78.8)	42 (56.8)	
No	49 (21.2)	32 (43.2)	
Arrival hour to ED			0.031
8 pm -20 am	150 (64.9)	58 (78.4)	
21 am-7 pm	81 (35.1)	19 (21.6)	
Site of pain			0.005
Abdominopelvic	77 (33.3)	21 (28.4)	
Musculoskeletal	47 (20.3)	13 (17.6)	
Thoracic	49 (21.2)	10 (13.5)	
Cephalic	22 (9.5)	20 (27)	
Lumbar	13 (5.6)	6 (8.1)	
Multifocal	23 (10)	4 (5.4)	
Mode of appearance			< 0.001
Sudden	129 (55.8)	20 (27)	
Progressive	102 (44.5)	54 (73)	
Duration of pain before admission (Hours),			< 0.001
<6	76 (32.9)	6 (8.1)	
6-72	75 (32.5)	22 (29.7)	
72-168	39 (16.9)	27 (36.5)	
>168	41 (17.7)	19 (25.5)	
Medication before admission			0.18
Yes	54 (23.4)	23 (31.1)	
No	177 (76.6)	51 (68.9)	
Delay of care (Hours),			0.08
<30	104 (45)	21 (28.4)	
30-60	71 (30.7)	31 (41.9)	
60-120	44 (19)	17 (23)	
>120	12 (5.2)	5 (6.8)	
Length of stay (Hours), median (IQR)	48 [48–72]	48 [24–72]	0.58

Length of stay was expressed as median and interquartile range (IQR); *ED*: emergency department; *IQR*: Interquartile range.

In multivariate analysis, factors associated with insufficient pain management were: accompanied patients (OR = 2.72, 95% CI = 1.28- 5.76, p = 0.009), pain as chief complaint (OR = 2.32, 95% CI = 1,25-4.31, p = 0.007), cephalic site of pain (OR = 6.28, 95% CI = 2.26-17.46, p <0.001), duration of pain before admission more than 72 hours (72-168 h (OR = 7.85, 95% CI = 3.13-25.30, p = 0.001), and >168 h (OR = 4.55, 95% CI = 1.77-14.90, p = 0.02). Predictor factors of unrelieved pain were reported in Table 3.

**Table 3:** **Predictor factors of unrelieved pain in Emergency Department**

Characteristics	OR	95% IC	p value
Accompanied patient			
No	1	---	---
Yes	2.72	1.28-5.76	0.009
Pain as chief complaint			
Yes	1	---	---
No	2.32	1.25-4.31	0.007
Site of pain			
Thoracic	1	---	---
Abdominopelvic	1.37	0.55-3.35	0.49
Musculoskeletal	1.18	0.45-3.19	0.75
Cephalic	6.28	2.26-17.46	<0.001
Lumbar	2.18	0.61-7.76	0.23
Multifocal	1.79	0.43-7.38	0.42
Duration of pain			
<6	1	---	---
6-72	4.12	1.35-11.13	0.12
72-168	7.85	3.13-25.30	0.001
>168	4.55	1.77-14.90	0.02

H-L test (x^2^ = 8.08, p = 0.43); ROC (AUR = 0.76, 95% CI = 0.73- 0.84). OR: odds ratio. 95% CI: confidence interval. 1: reference category; *H*-*L*: Hosmer-Lemeshow; x^2^: Chi-square test. *AUC*: area under ROC curve; *ROC*: receiver-operating characteristics.

## Discussion

This study represents the first Moroccan survey of ED patient pain experience, and one of the very few studies which investigate pain relief among a sample of consecutive non-homogenous patients attending the ED during 10 days, 24 hours each day, and hospitalized for a short stay in ED [10]. The relief of pain in a non-homogenous patient during short stay in ED depend both analgesic use, patient differences about other associated injuries, and system factors.

This study reported high levels of intense to severe pain at ED arrival (91.1%), and a decrease in pain intensity at discharge (46.9%). However, only one quarter patients felt on discharge from the ED that their pain had not been relieved, this rate is very lower than those report from previous study [2, 3, 10, 21]. We report a relatively high rate of analgesic use. Though, there is a wide discrepancy in the literature concerning the rate of analgesic uses in the ED, ranging from 40% to 78% [4, 5, 22–24]. Unrelieved acute pain and persistent pain is a widespread global problem for divergent patient group across the lifespan [25, 26]. However, in this study, the finding of a high incidence of relief of pain in Rabat ED is a positive, and encouraging outcome, that puts the ED in a positive light, at least from the point of view of managing pain.

The predictors factors of unrelieved pain were accompanied patients, pain as chief complaint, the cephalic site of pain, and longuer duration of pain before ED admission.

The ED is a specific environment where cultural clashes are common among the patients and providers and among patients and their families [27]. Companions may facilitate, inhibit, or impede patient participation and autonomy. Family unfamiliarity with the health care system, lack of insurance, and intolerance to painfully long waiting times make them so frustrated that even the theoretical possibility of timely, efficient, and adequate pain management seems unrealistic [27, 28].

In this study, when the pain was the main reason for ED visit, it is better relieved. Acute pain is protective and useful because it is warning, and allows safeguard of the organism integrity. Therefore, within the emergency care setting, somatic problems take priority over the control of acute pain [3].

Yet when the pain is cephalic, it is less relieved. Medical or surgical origin of the pain does not been noted in this study. Unrelieved pain, would be linked to a lack of clinical exam which should objectified pain; lack of systematic assessment of pain by physicians, lack of complaint from the patient. When the neurological investigations are not contributing, maybe the physician does not treat anything, even pain and started further investigations, for etiological research without worrying about of the pain symptoms. This is unlikely to occur until pain is adequately addressed and treated appropriately as a true emergency.

Unrelieved pain was also related to a longer time before the ED admission [1, 10, 29]. In effect, pain produces a physiological stress response that includes increased heart and breathing rates to facilitate the increasing demands of oxygen and other nutrients to vital organs. Failure to relieve pain produces a prolonged stress state, which can result in harmful multisystem effects [29–32]. A number of recent surveys, supported by the Canadian Pain Society, have indicated that there are very long waiting times for treatment; on average, several weeks or months. This is particularly serious, as it has been documented that if acute pain or injury is not treated effectively early on, it can develop into a chronic pain condition and become worse and worse if it is not treated properly [15, 31–33].

Our study may have underestimated the prevalence of pain in the ED population. Many patients including those who are unconscious, intubated, seriously ill or injured patients and those with speech impairments and language barriers may have been unable to communicate their pain. Other patients may have had a latency of onset of pain and not experienced pain at the time of their ED visit.

There are some limitations to our study. First, our study was conducted in a large urban teaching hospital in Morocco, which may not be representative of other emergency care settings. Second, our study was conducted during 10 days, this short duration may under-represent the number of injuries. Another limitation of all pain prevalence studies is the inherent problem of categorizing and even defining pain. There are many aspects of pain classification in which consensus is lacking. For example, there is no one accepted definition for chronic pain. Chronic pain has been defined as pain that persists usually for 6 months or more and no longer signals real or impending tissue damage. However, as Turk and Okifuji noted, the 2 most commonly used chronologic markers used to denote chronic pain have been 3 months and 6 months since the initiation of pain; however, these distinctions are arbitrary” [18, 34]. Bonica referred to the language ambiguity of pain classification as a “modern tower of Babel” [35].

The finding of a high incidence of relief of pain in this study is a positive and encouraging result, which puts the ED in a positive light, at least from the point of view of managing pain. Comprehensive pain assessment and management are essential to reduce the prevalence and burden of pain, and new strategies are required to support these changes [24]. Given the variability among countries in health care policies and programs, resources and educational programs, many of the approaches and strategies outlined will need to be tailored to each country’s socioeconomic and educational situation [15]. In providing effective care to the populations served by the ED healthcare workers, we have a great responsibility to relieve pain by all possible appropriate means in a timely, efficient and effective manner. Albert Schweitzer once said, “We must all die. But that I can save a person from days of torture that is what I feel is my great and ever-new privilege. Pain is a more terrible lord of mankind than even death itself”.
